# Thiazolo[3,2-*a*]benzimidazoles: Synthetic Strategies, Chemical Transformations and Biological Activities

**DOI:** 10.3390/molecules15063775

**Published:** 2010-05-26

**Authors:** Khalid A. Al-Rashood, Hatem A. Abdel-Aziz

**Affiliations:** Department of Pharmaceutical Chemistry, College of Pharmacy, King Saud University, P.O. Box 2457, Riyadh 11451, Saudi Arabia

**Keywords:** thiazolo[3,2-*a*]benzimidazole, thiazolo[3,2-*a*]bezimidazol-3(2*H*)one, YM-298198, tilomisole, anticancer activities, MGlu1 antagonists

## Abstract

The present review covers the recent synthetic strategies and chemical transformations of thiazolo[3,2-*a*]benzimidazoles and it also presents the highlights of the biological activities of these compounds.

## 1. Introduction

**Figure 1:**
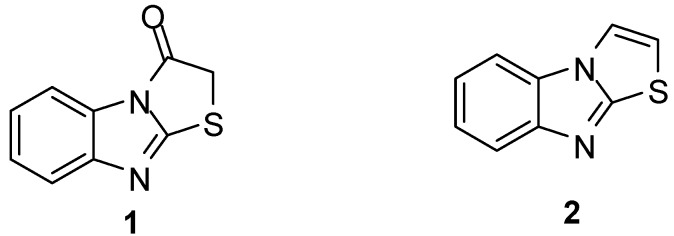
Structure of compounds **1** and **2**.

Thiazolo[3,2-*a*]benzimidazole systems have been known for more than seven decades [1]. Thiazolo[3,2-*a*]bezimidazol-3(2*H*)one (**1**, Figure 1)) was synthesized in 1926, while thiazolo[3,2-*a*]-benzimidazole (**2**, Figure 1) was reported in 1966 [2,3]. Nevertheless, various substituted thiazolo[3,2-*a*]-benzimidazoles were reported before compound **2** [4,5,6,7,8]. 

In 1988, a review on fused thiazolobenzimidazoles was published by Chimirri *et al.* [9]. In recent years, there has been considerable interest of researchers in thiazolo[3,2-*a*]benzimidazoles, stimulated by their biological activities. Additionally an enormous variety of thiazolo[3,2-*a*]-benzimidazoles with unique pharmaceutical and medicinal applications have been reported in the patent literature. These considerable biological activities and our contributions to the chemistry and biological activities of these compounds prompted us to compile the present review which deals with the recent synthetic strategies, chemical transformations and biological activities of thiazolo[3,2-*a*]benzimidazoles. The intention of this review is to focus mainly on publications appeared from 1989 to the end of 2009.

## 2. Synthetic Strategies

### 2.1. From 2-mercaptobenzimidazoles

**Scheme 1:**
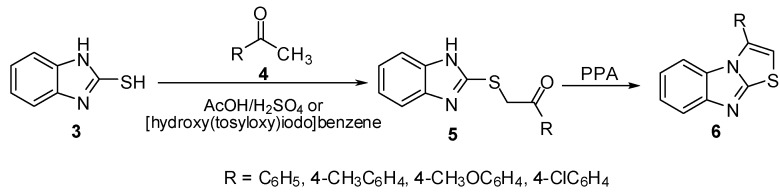
Reaction of 2-mercaptobenzimidazole (**3**) with ketones **4**.

2-Mercaptobenzimidazoles were used in the synthesis of several thiazolo[3,2-*a*]benzimidazole derivatives by annulations of thiazole ring to a benzimidazole moiety. The reaction of 2-mercapto-benzimidazole (**3**) with ketones **4** in boiling AcOH/H_2_SO_4_ afforded 2-benzimidazolylthioacetophenone derivatives **5** [10,11,12]. The latter sulphides were cyclized to give the corresponding thiazolo[3,2-*a*]benzimidazoles **6** using PPA or [hydroxy(tosyloxy)iodo]benzene (Scheme 1).

**Scheme 2:**
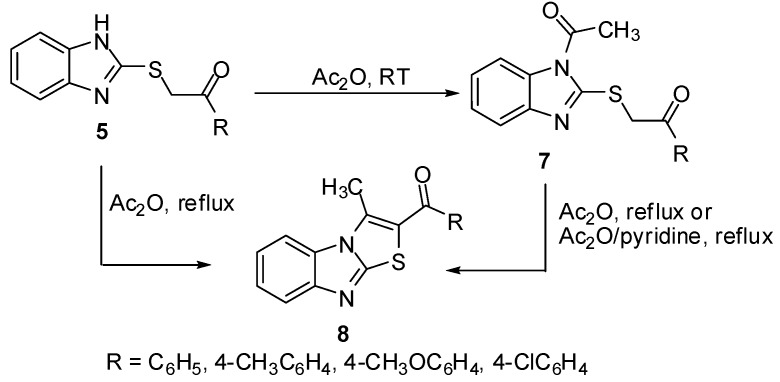
Cyclization of sulphides **5**.

Treatment of sulphides **5** with acetic anhydride at room temperature gave the *N*-acetyl derivatives **7**. On the other hand, heating of thioacetophenones **5** in acetic anhydride or in Ac_2_O/pyridine mixture afforded the 2-aroyl-3-methylthiazolo[3,2-*a*]benzimidazoles **8 **[11] which were obtained independently by refluxing *N*-acetyl derivatives **7** in Ac_2_O (Scheme 2). 

**Scheme 3:**
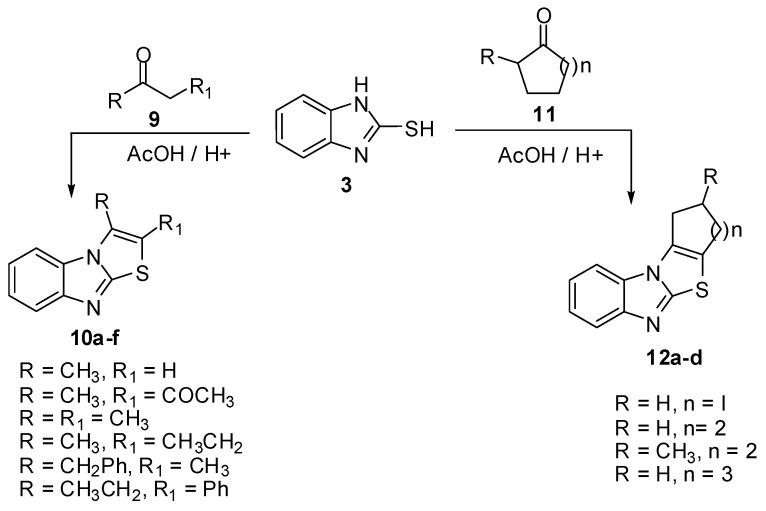
Reaction of 2-mercaptobenzimidazole (**3**) with ketones **9 **and **11**.

The reaction of 2-mercaptobenzimidazole (**3**) with aliphatic ketones **9a-f **[11] such as acetone, acetylacetone, butanone, pentan-2-one and 1-phenylbutan-2-one using acidified acetic acid gave the corresponding thiazolo[3,2-*a*]benzimidazoles **10a-f** in good yield. Alicyclic ketones **11a-d** like cyclopentanone, cyclohexanone, 2-methylcyclohexanone and cycloheptanone were allowed to react with 2-mercaptobenzimidazole (**3**) under the same reaction conditions to obtain the tetracyclic compounds **12a-d **[11] (Scheme 3).

**Scheme 4:**
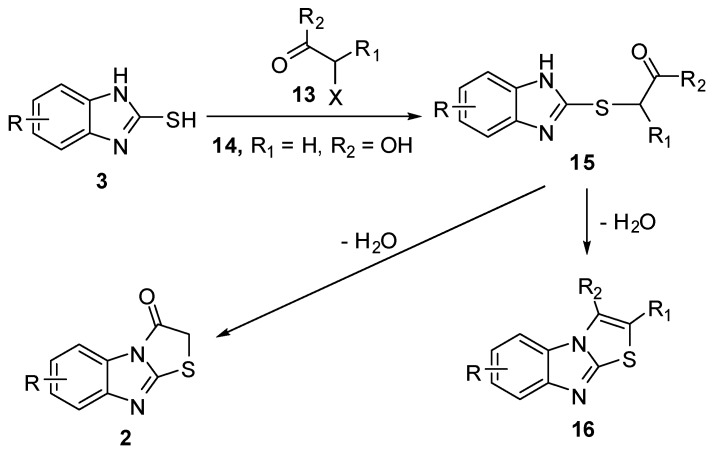
Reaction of 2-mercaptobenzimidazoles **3** with α-halo ketones **13**.

Thiazolo[3,2-*a*]benzimidazoles **2 **and **16 **[13,14,15,16,17,18,19,20,21,22,23,24,25,26,27] were obtained by the reaction of 2-mercaptobenzimidazoles **3** with various α-halo ketone derivatives **13 (14)** which gave the corresponding acyclic intermediates **15**. Cyclization of the latter by acetic anhydride/pyridine mixture, polyphosphoric acid or sodium ethoxide gave compounds **16** (Scheme 4). Cyclization of 5-substituted-(2-benzimidazolyl)thioacetic acid **15** (R_1_ = H, R_2_ = OH) led to the formation of two isomers with the substituent in 6 or 7 position as established through NMR analysis of the reaction products [25,27] (Scheme 4).

**Scheme 5:**
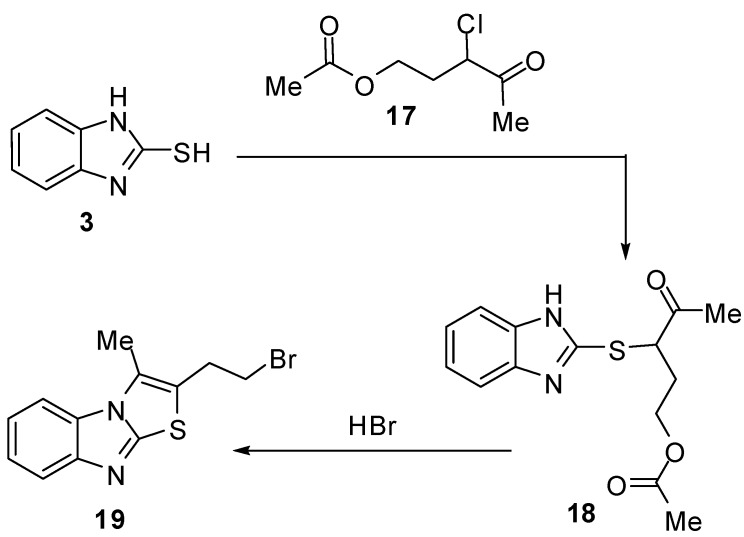
Reaction of 2-mercaptobenzimidazoles (**3**) with α-halo ketone **17**.

Interaction of 2-mercaptobenzimidazole (**3**) with 3-chloro-3-acetopropyl acetate (**17**) [28] with subsequent cyclization of the intermediate 3-acetyl-1-acetoxypropylmercaptobenzimidazole (**18**) *via* hydrobromic acid afforded 2-bromoethyl-3-methylthiazolo[3,2-*a*]benzimidazole **19** (Scheme 5).

**Scheme 6:**
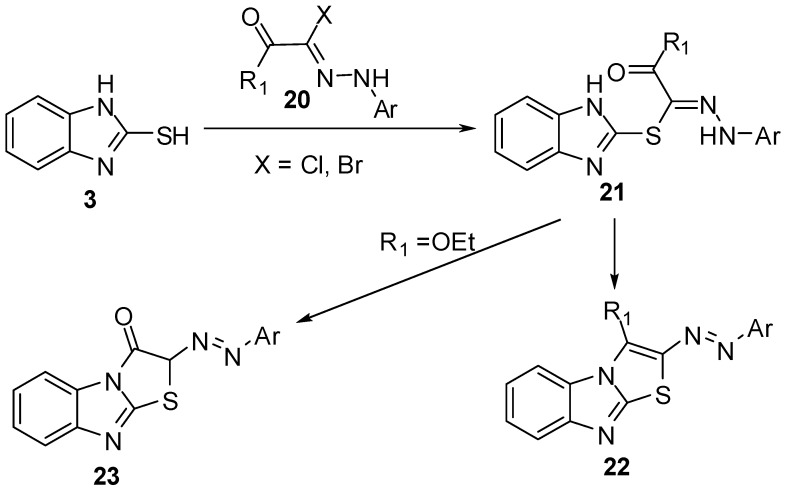
Reaction of 2-mercaptobenzimidazole (**3)** with hydrazonyl halides **20**.

2-Hydrazonolthiazolo[3,2-*a*]benzimidazoles **22 **and **23 **[29,30,31,32,33] were prepared by the reaction of 2-mercaptobenzimidazole (**3**) with hydrazonyl halides **20** followed by cyclization of hydrazones **21**(Scheme 6).

**Scheme 7:**
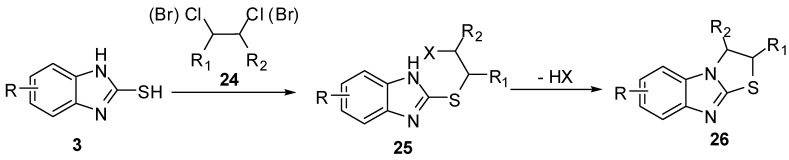
Reaction of 2-mercaptobenzimidazoles **3** with 1,2-dihaloethyl derivatives **24**.

The reaction of 2-mercaptobenzimidazoles **3** with 1,2-dihaloethyl derivatives **24** [34,35,36] in the presence of basic reagents, affords 2-(*β*-haloethylthio)benzimidazole **25**. Cyclization of the latter intermediate gives 2,3-dihydrothiazolo[3,2-*a*]benzimidazoles **26**(Scheme 7).

**Scheme 8:**
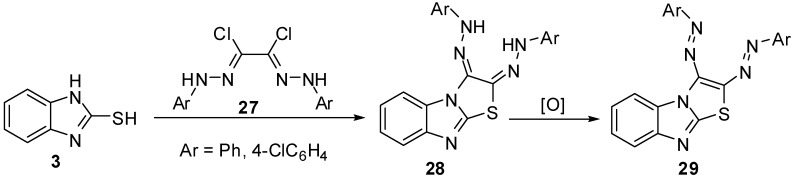
Reaction of 2-mercaptobenzimidazole (**3)** with bis-hydrazonoyl chlorides **27**.

2,3-(Diaryl)hydrazono-2,3-dihydrothiazolo[3,2-*a*]benzimidazoles **28** [37,38] were synthesized *via* the reaction of bis-hydrazonoyl chlorides **27 **with 2-mercaptobenzimidazole (**3**). Oxidation of the latter hydrazones resulted in the formation of 2,3-diazothiazolo[3,2-*a*]benzimidazoles **29 **(Scheme 8).

**Scheme 9:**
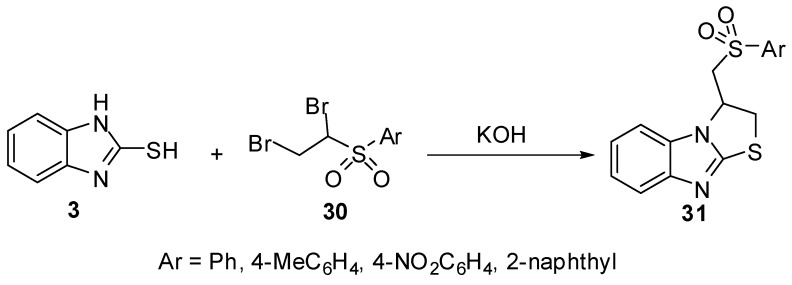
Reaction of 2-mercaptobenzimidazole (**3)** with 1,2-dibromoethylsulfonyles **30**.

Additionally, arylsulphones **31 **[39] were prepared by heterocyclization reaction of 1,2-dibromoethylsulfonyles **30** with 2-mercaptobenzimidazole (**3**) in presence of potassium hydroxide (Scheme 9).

**Scheme 10:**
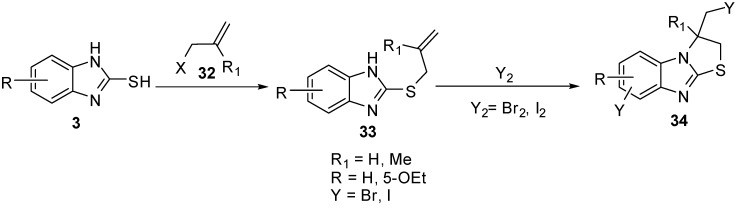
Reaction of 2-mercaptobenzimidazoles **3** with allyl halides **32**.

2-(Allylthio)-1*H*-benzimidazoles **33** were prepared by the reaction of 2-mercaptobenzimidazole (**3**) with allyl halides **32**. Cyclization of **33** with iodine or bromine [40,45] gives 3-(halomethyl)-2,3-dihydro-3-methyl-thiazolo[3,2-*a*]benzimidazole derivatives **34**(Scheme 10). The bromination of **33** 5-ethoxy-2-alkenylthiobenzimidazole (R = -OEt) proceeds on the C6 site of benzimidazole ring in parallel with heterocyclization [45] (Scheme 10). 

**Scheme 11:**
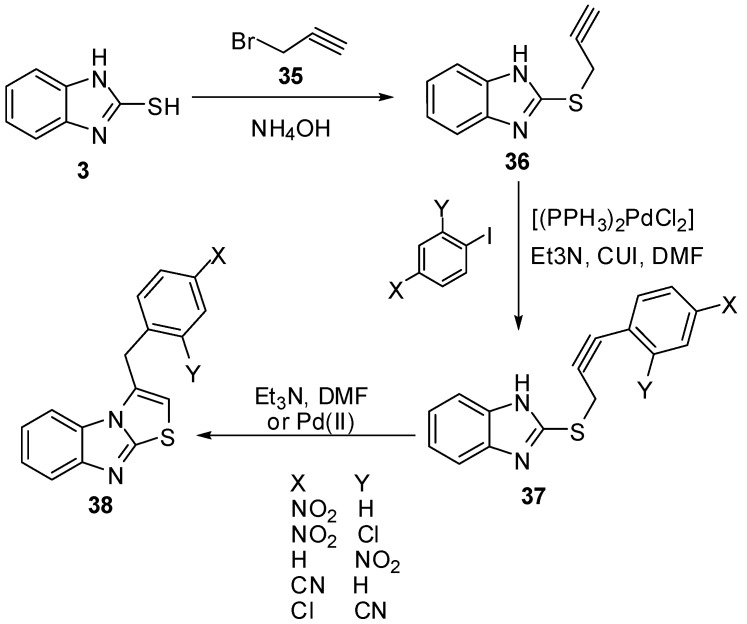
Reaction of 2-mercaptobenzimidazole (**3)** with propargyl bromide (**35**).

2-Mercaptobenzimidazole (**3**) was reacted with propargyl bromide (**35**) in refluxing EtOH in the presence of NH_4_OH to yield 2-propargylmercaptobenzimidazole (**36**) [46]. When **36** was treated in DMF with aryl halides and triethylamine in the presence of bis(triphenylphosphine)palladium chloride and copper iodide at room temperature, 3-benzylthiazolo[3,2-*a*]benzimidazoles **38** were obtained *via* the intermediate **37** (Scheme 11).

**Scheme 12:**
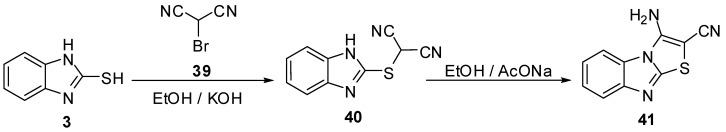
Reaction of 2-mercaptobenzimidazole (**3)** with bromomalononitrile (**39**).

3-Aminothiazolo[3,2-*a*]benzimidazol-2-carbonitrile (**41**) was prepared by the reaction of 2-mercabtobenzimidaziole (**3) **with bromomalononitrile (**39**) in ethanol followed by cyclization reaction of product **40***via* anhydrous sodium acetate [11,47,48] (Scheme 12).

**Scheme 13:**
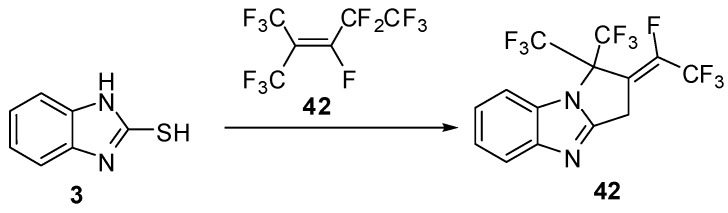
Reaction of 2-mercaptobenzimidazole (**3)** with fluoro ethylene derivative **42**.

The reaction of 2-mercaptobenzimidazole (**3**) with fluoro ethylene derivative **42 **[49] gives the functionally fluoro thiazolo[3,2-*a*]benzimidazole derivatives **43** (Scheme 13).

**Scheme 14:**
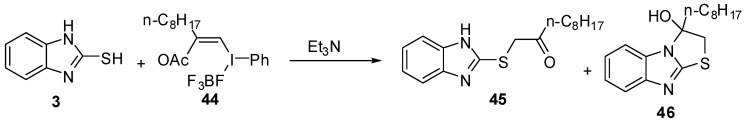
Reaction of 2-mercaptobenzimidazole (**3)** with iodanes **44**.

Recently, Masahito *et al.* [50] reported the reaction of 2-mercabtobenzimidazole (**3**) with (*Z*)-(2-acetoxy-1-decenyl)phenyl-λ^3^-iodanes (**44**) to afford the *α*-thio ketone **45** in equilibrium with the cyclized alcohol **46**(Scheme 14).

**Scheme 15:**
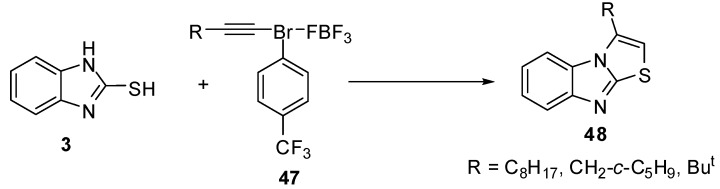
Reaction of 2-mercaptobenzimidazole (**3)** with bromanes **47**.

Exposure of 1-decynyl-λ^3^-bromane **47a** to benzimidazole **3** resulted in the formation of 3-octylthiazolo[3,2-*a*]benzimidazole (**48a**). Similar results were obtained by the reactions of 3-(cyclopentyl)-1-propynyl **47b** and 3,3-dimethyl-1-butynyl-3-bromane **47c** to produce 3-(cyclopentylmethyl)- (**48b**) and 3-tert-butylthiazolo[3,2-*a*]benzimidazole (**48c**) [51] (Scheme 15).

**Scheme 16:**
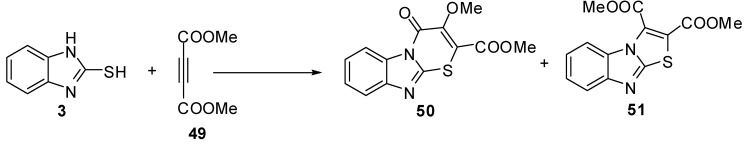
Reaction of 2-mercaptobenzimidazole (**3)** with dimethyl acetylenedicarboxylate (**49)**.

Moreover, the reaction of compound **3** with dimethyl acetylenedicarboxylate (**49) **gave 3-methoxy-4-oxo-4*H*-1-thia-4*a*,9-diaza-fluorene-2-carboxylic acid methyl ester (**50**) and benzo[4,5]imidazo[2,1-*b*]thiazole-2,3-dicarboxylic acid dimethyl ester (**51**) [52] as shown in Scheme 16.

**Scheme 17:**
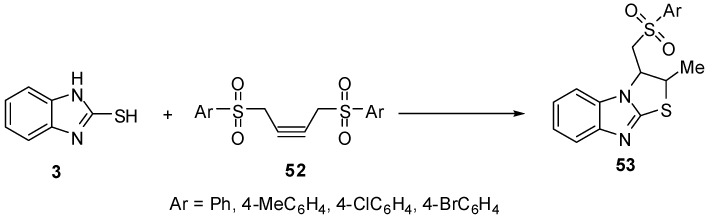
Reaction of 2-mercaptobenzimidazole (**3)** with 1,4-diarylsulfonyl-2-butynes **52**.

Treatment of 2-mercaptobenzimidazole (**3) **with 1,4-diarylsulfonyl-2-butynes **52 **gives 2,3-dihydro-3-[(arylsulfonyl)methyl]-2-methylthiazolo[3,2-*a*]benzimidazoles **53 **[53] (Scheme 17).

**Scheme 18:**
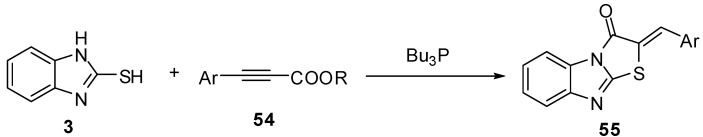
Reaction of 2-mercaptobenzimidazole (**3)** with arylpropiolates **54**.

2-Arylidinethiazolo[3,2-*a*]benzimidazol-3(2*H*)-ones **55** were developed using a phosphine-catalyzed tandem addition and intramolecular cyclization of 2-mercaptobenzimidazole (**3**) on arylpropiolates **54 **[54] (Scheme 18).

**Scheme 19:**
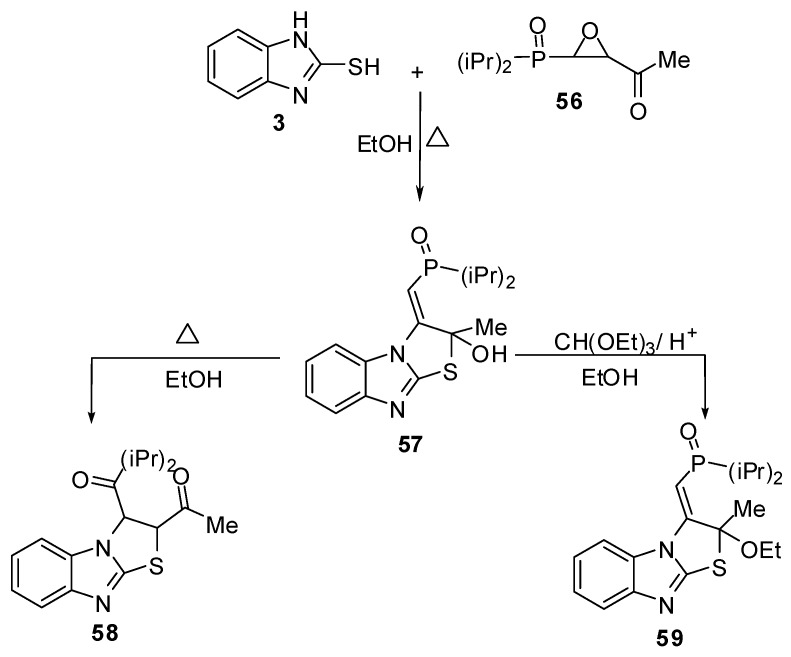
Reaction of 2-mercaptobenzimidazole (**3)** with epoxyphosphorus derivative **56**.

Treatment of compound **3 **with the epoxyphosphorus derivative **56 **[55] in ethanol gives isolable intermediate **57**. Rearrangement of 2,3-dihydrothiazolo[3,2-*a*]benzimidazole **57** takes place in ethanol to give the acetyl derivative **58**, while the reaction of compound **57** with triethyl orthoformate in acidic medium afforded the ethoxy derivative **59** (Scheme 19).

**Scheme 20:**
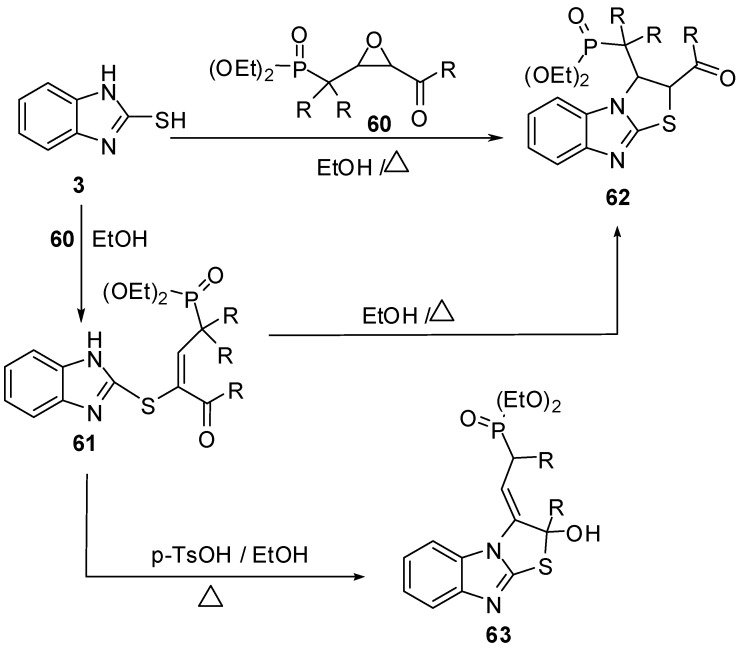
Reaction of 2-mercaptobenzimidazole (**3)** with epoxide derivative **60**.

Furthermore, 2-mercaptobenzimidazole (**3**) reacts with epoxide derivative **60 **to give compound **61** which cyclized in refluxing ethanol to give 2,3-dihydrothiazolo[3,2-*a*]benzimidazole derivative **62**, or in the presence of p-toluenesulfonic acid to give compound **63**. Compound **62 ** prepared directly by the reaction of compound **3 **with epoxide **54** in refluxing ethanol [55] (Scheme 20).

**Scheme 21:**
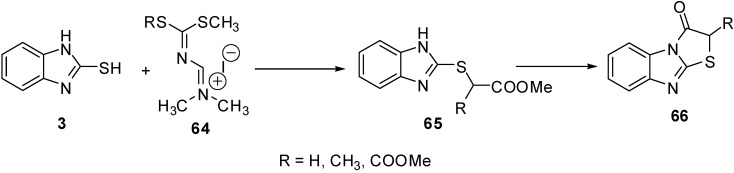
Reaction of 2-mercaptobenzimidazole (**3)** with amidinium salts **64**.

A series of 2-*R*-thiazolo[3,2-*a*]benzimidazol-3(2*H*)-ones **66 **[56] were prepared from the intermediate **65.** The latter intermediate was prepared by the reaction of amidinium salts **64** and 2-mercapto-benzimidazole (**3**) (Scheme 21).

### 2.2. From 1-alkylbenzimidazoles

**Scheme 22:**
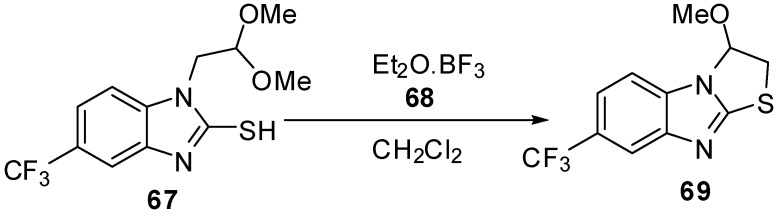
Reaction of compound **67** with diethyl ether-boron trifluoride **68**.

The interamolecular cyclization of 1-(dimethoxyethyl)-2-mercaptobenzimidazole derivative **67 **[57] by diethyl ether-boron trifluoride **68** in dry methylene chloride furnished 2,3-dihydrothiazolo[3,2-*a*]benzimidazole derivative **69 **(Scheme 22).

**Scheme 23:**
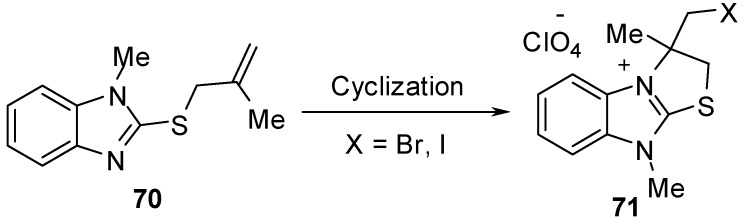
Cyclization of compound **70**.

The cyclization reaction of compound **70 **[58,59] gives 2,3-dihydro-3-(halomethyl)-3,9-dimethylthiazolo[3,2-*a*]benzimidazolium perchlorate derivatives **71** (Scheme 23). 

### 2.3. From 2-chlorobenzimidazoles

**Scheme 24:**
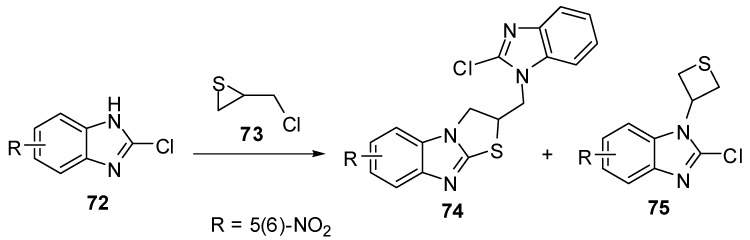
Reaction of benzimidazole **72** with (chloromethyl)thiirane (**73**).

Treatment of 2-chloro-5(6)-nitrobenzimidazole **72** with (chloromethyl)thiirane (**73**) [60,61]afforded a mixture of 2,3-dihydro-2-[(2-chlorobenzimidazol-1-yl)methyl]thiazolo[3,2-*a*]-benzimidazoles **74** and 2-chloro-1-(3-thietanyl)benzimidazoles **75**(Scheme 24).

### 2.4. From 1,3-thiazoles

**Scheme 25:**
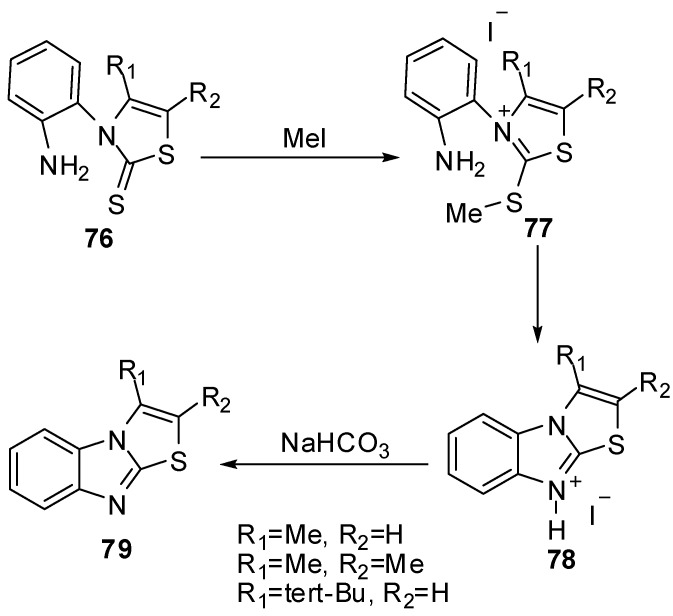
Reaction of thiazoline-2-thione **76** with methyl iodide.

*N*-(2-Aminophenyl)-thiazoline-2-thione **76** reacted with methyl iodide in acetone at room temperature to afford quantitatively the thiazolium iodides **77** [62]. The latter thiazolium iodides were refluxed in methanol to afford thiazolo[3,2-*a*]benzimidazolium iodides **78** which treated with NaHCO_3_ to afford thiazolo[3,2-*a*]benzimidazole derivatives **79** (Scheme 25).

### 2.5. From other reagents

3-Aryl-2-*N*,*N*-dimethylaminothiazolo[3,2-*a*]benzimidazoles were prepared from diisothiocyanates [63], 2,3-dihydrothiazolo[3,2-*a*]benzimidazole of platinum amine complexes were also prepared [64]. Regioselective synthesis of 2-methoxy carbonyl-thiazolo[3,2-*a*]benzimidazole-6(7)-carboxylic acid were reported using crystallization induced region-isomerization [65]. 

## 3. Chemical Transformations

In this part, each sub-title was specified for the reaction(s) of certain atom and/or its substiuent(s) in thiazolo[3,2-*a*]benzimidazole ring system. *Chemical Abstract* numbering of thiazolo[3,2-*a*]benzimidazole atoms was considered.

Figure 2

**Figure 2:**
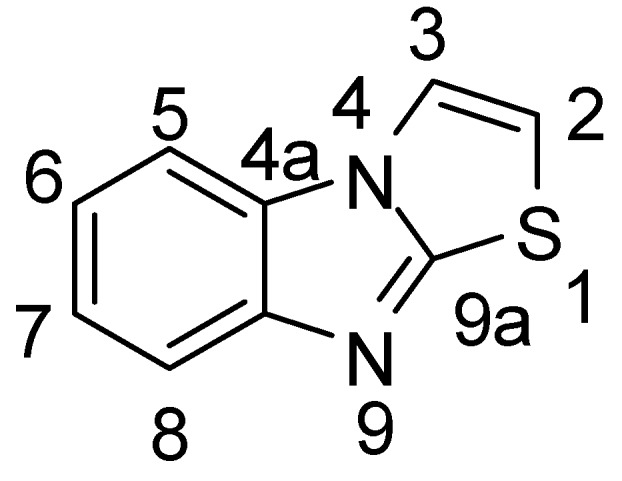
*Chemical Abstract* numbering of thiazolo[3,2-*a*]benzimidazole atoms.

### 3.1. Reactions of S1

**Scheme 26:**
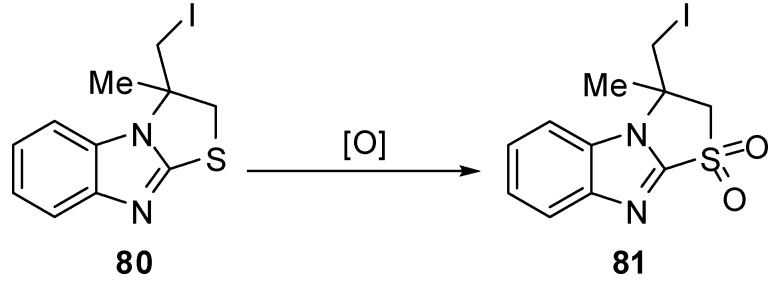
Oxidation of 2,3-dihydro-1,1-dioxthiazolo[3,2-*a*]benzimidazole **81**.

2,3-Dihydro-1,1-dioxthiazolo[3,2-*a*]benzimidazole **81** [66] was synthesized by oxidation of the corresponding thiazazolo[3,2-*a*]benzimidazole **80** with hydrogen peroxide in the presence of K_2_WO_4_ under mild conditions (Scheme 26).

**Scheme 27:**
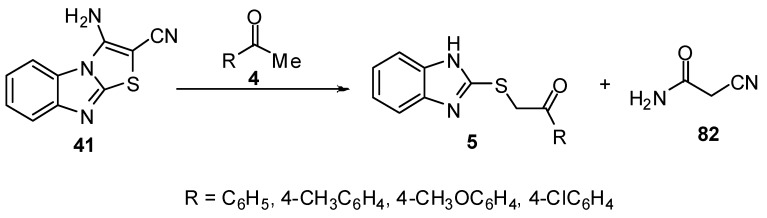
Reaction of 3-aminothiazolo[3,2-*a*]-benzimidazole-2-carbonitrile (**41**) with ketones **4**.

2-Benzimidazolylthioacetophenones **5 **[11] were obtained when 3-aminothiazolo[3,2-*a*]-benzimidazole-2-carbonitrile (**41**) was allowed to react with aromatic ketones **4** using acidified acetic acid (Scheme 27). 

### 3.2. Reactions of S1-C1

**Scheme 28:**
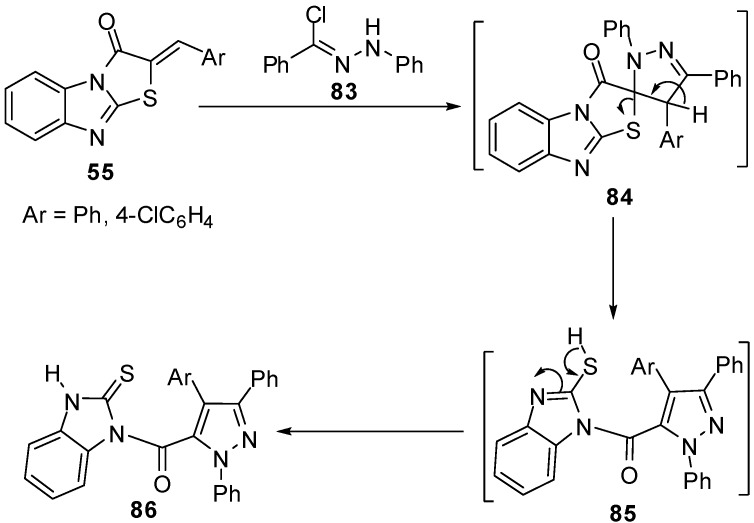
Reaction of 2-arylidenethiazolo[3,2-*a*]benzimidazol-3(2*H*)-one **55** with nitrilimine **83**.

Microwave irradiation has been applied for a rapid and efficient synthesis of 2-arylidene-thiazolo[3,2-*a*]benzimidazol-3(2*H*)-ones **55 **from 2-mercapto-1*H*-benzimidazole (**3**) [67]. Abdel-Aziz *et al.* reported the reaction of nitrilimine **83** with 2-arylidenethiazolo[3,2-*a*]benzimidazol-3(2*H*)-one **55** to afford pyrazoles **84**. Spectroscopic analyses confirmed the regioselective 1,3-dipolar cycloaddition of the nitrilimine **83** to the exocyclic double bond of **55** to afford non-isolable spiro intermediate **84** which rearranged to the corresponding pyrazolylbenzimidazole derivatives **86 **as shown in Scheme 28 [67].

### 3.3. Reactions of S1-C9a

**Scheme 29:**
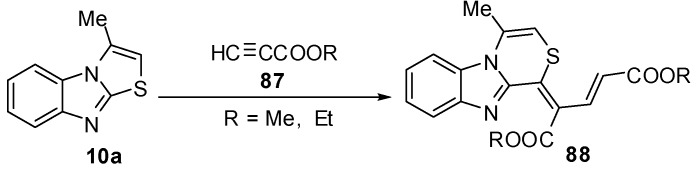
Reaction of 3-methylthiazolo[3,2-*a*]benzimidazole (**10a**) with propiolic esteres **87**.

Abe *et al.* reported a re-investigation for the reaction of 3-methylthiazolo[3,2-*a*]benzimidazole (**10a**)with propiolic esteres **87 **[68] which gave the thiazino[4,3-*a*]-benzimidazole rearrangement product derivative **88** (Scheme 29).

### 3.4. Reactions of C2

#### 3.4.1. Reactions of 3-methylthiazolo[3,2-*a*]benzimidazole (10a)

**Scheme 30:**
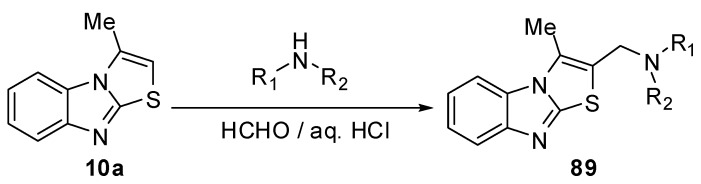
Mannich reaction of 3-methylthiazolo[3,2-*a*]benzimidazole **10a**.

Mannich reaction of 3-methylthiazolo[3,2-*a*]benzimidazole **10a** with some secondary amines and paraformaldehyde in aqueous HCl gave the corresponding Mannich bases **89 **[69] (Scheme 30).

#### 3.4.2. Reactions of 1-(3-methylthiazolo[3,2-*a*]benzimidazol-2-yl)ethanone (**10b**)

**Scheme 31:**
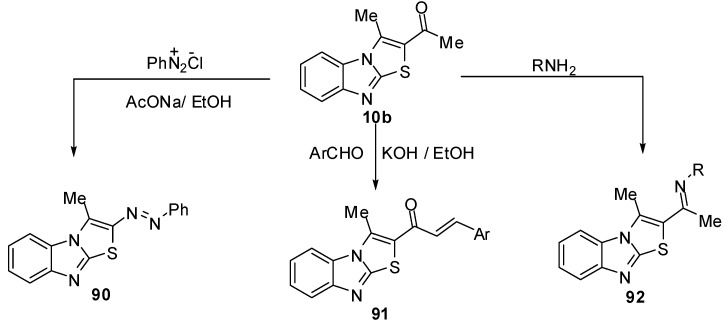
Reactions of ethanone derivative **10b**.

1-(3-Methylthiazolo[3,2-*a*]benzimidazol-2-yl)ethanone (**10b**) reacts with the aniline diazonium chlorideto afford 3-methyl-2-(phenylazo)thiazolo[3,2-*a*]benzimidazole (**90**) [13] (Scheme 31).

Moreover, condensation of **10b** with some aromatic aldehydes in ethanolic potassium hydroxide gave the corresponding 2-(3-aryl-1-oxo-2-propenyl)-3-methylthiazolo[3,2-*a*]benzimidazole **91**. The reactions of ethanone **10b** with hydroxylamine, methyl amine or ethyl amine were reported [13] (Scheme 31).

**Scheme 32:**
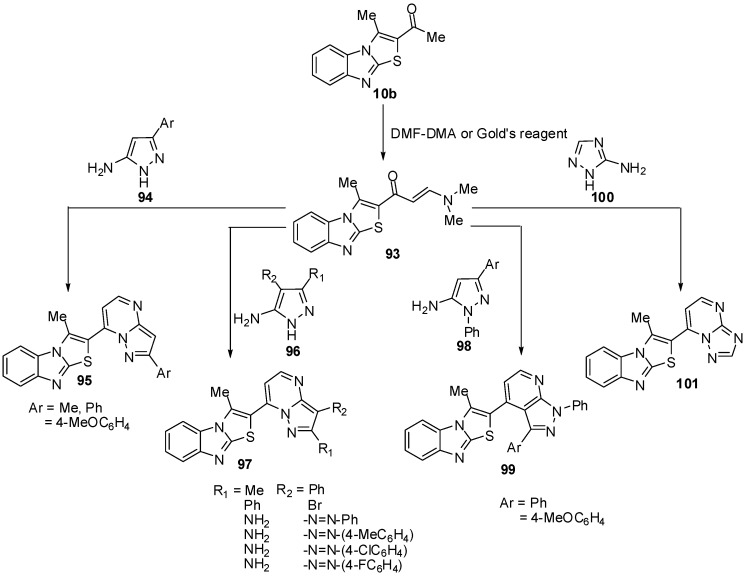
Reaction of ethanone **10b** with DMF-DMA.

1-(3-Methylthiazolo[3,2-*a*]benzimidazol-2-yl)ethanone (**10b**) was treated with dimethylformamide-dimethylacetal (DMF-DMA), in dry xylene, at reflux temperature, it afforded *E*-3-(*N*,*N*-dimethylamino)-1-(3-methylthiazolo[3,2-*a*]benzimidazol-2-yl)prop-2-en-1-one (**93**) [70] (Scheme 32). Recently, Abdel-Aziz *et al.* reported an alternative synthesis of compound **93** using [3-(dimethyl-amino)-2-azaprop-2-en-1-ylidene]dimethylammonium chloride (Gold’s reagent) [71] where Gold's reagent reacted with ketone **10b** in sodium methoxide to produce the enaminone **93** (Scheme 32). The reaction of compound **93** with aminopyrazoles **94** in refluxing pyridine [70] or in acetic acid presence of H_2_SO_4 _[71] afforded pyrazolo[1,5-*a*]pyrimidines **95**. 

In a similar manner, treatment of compound **93 **with 5-amino-3-methyl-4-phenyl-1*H*-pyrazole (**96a**), 4-bromo-5-amino-3-phenyl-1*H*-pyrazole (**96b**) or 4-(arylhydrazono)-3,5-diamino-1*H*-pyrazole **96c-f** resulted in the formation of pyrazolo[1,5-*a*]pyrimidines **97a**, **97b** and **97c-f**, respectively [71] (Scheme 32). Next, the reaction of enaminone **93 **with 5-amino-3-aryl-1*-*phenylpyrazole **98a**,**b** in refluxing glacial acetic acid, in the presence of sulphuric acid, yielded the corresponding pyrazolo[3,4-*b*]pyridine derivatives **99a,b** (Scheme 32). When compound **93** was treated with 3-amino-1,2,4-(1*H*)-triazole (**100**) in refluxing pyridine, it furnished the 1,2,4-triazolo[1,5-*a*]pyrimidine derivative **101** (Scheme 32) [70].

**Scheme 33:**
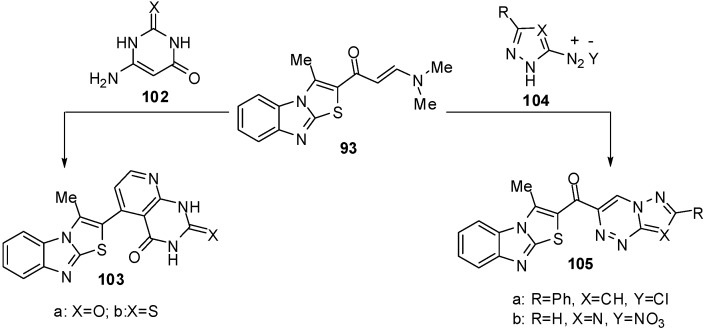
Reactions of enaminone **93**.

Furthermore, the reaction of enaminone **93** with 6-amino-1*H*-pyrimidin-2,4-dione (**102a**) or 6-amino-2-thioxo-2,3-dihydro-1*H*-pyrimidin-4-one (**102b**) in refluxing acetic acid resulted in the formation of pyrido[2,3-*d*]pyrimidines **103a** and **103b** (Scheme 33). The enaminone **93** treated with the diazonium salt of 3-phenyl-5-amino-1*H*-pyrazole **104a** or 5-amino-l,2,4-(1*H*)-triazole **104b** to afford non-isolable azo-coupling intermediates which cyclized *via* dimethylamine elimination yielded the pyrazolo[5,1-*c*]-l,2,4-triazine and l,2,4-triazolo[5,1-*c*]-l,2,4-triazine derivatives **105a** and **105b** (Scheme 33) [70].

**Scheme 34:**
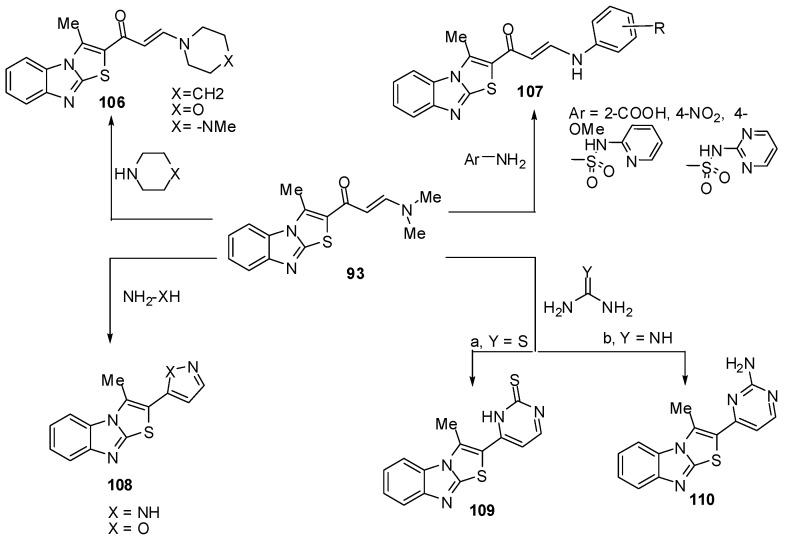
Reactions of enaminone **93**.

The enaminone **93** treated with some secondary amines like piperidine, morpholine or 1-methylpiperazine in refluxing ethanol to afford the corresponding tertiary amines **106**, respectively (Scheme 34) while treatment of **93** with anilines, sulphapyridine and sulphapyrimidine in refluxing acetic acid gave the corresponding acyclic secondary amine derivatives **107 **[72] (Scheme 34). The enaminone **93** reacted with hydrazine to afford 3-methyl-2-(2*H*-pyrazol-3-yl)thiazolo[3,2-*a*]benzimidazole (**108a**) while its reaction with hydroxylamine gave 2-(isoxazol-5-yl)-3-methylthiazolo[3,2-*a*]benzimidazole (**108b**) (Scheme 34). 

Furthermore, treatment of compound **93** with thiourea in refluxing ethanol, in the presence of sodium ethoxide, afforded pyrimidine-2-thione derivative **109**. It reacted also with guanidine to give the corresponding pyrimidine derivative **110** (Scheme 34) [72].

**Scheme 35:**
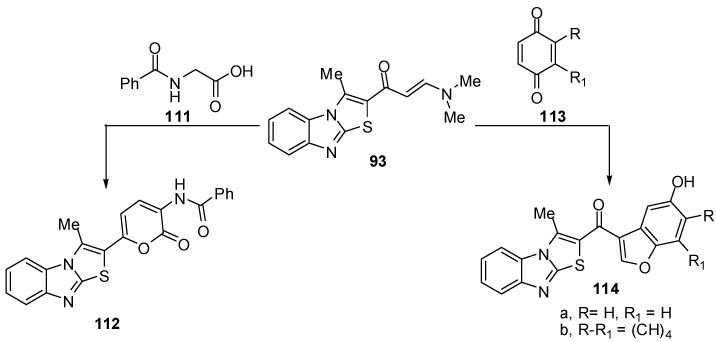
Reactions of enaminone **93**.

Treatment of compound **93** with 2-benzamidoacetic acid (**111**) in refluxing acetic anhydride to yield N-[6-(3-methylthiazolo[3,2-*a*]benzimidazol-2-yl)-2-oxo-2*H*-pyran-3-yl]benzamide (**112**) [72] (Scheme 35). Treatment of the enaminone **93** with *p*-benzoquinone (**113a**) in acetic acid at room temperature, afforded the benzo[*b*]furan derivative **114a**. In a similar manner, the enaminone **93** reacted with 1,4-naphthoquinone (**113b**) and afforded 2-(5-hydroxy-naphtho[1,2-*b*]furan-3-oyl)-3-methylthiazolo[3,2-*a*]benzimidazole (**114b**) [72] (Scheme 35).

**Scheme 36:**
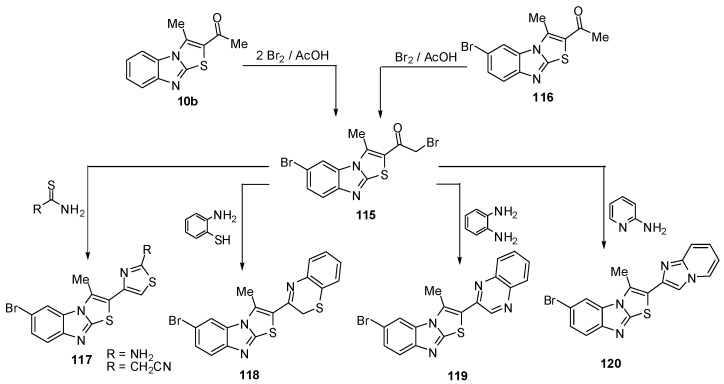
Bromination of ethanone **10b**.

The reaction of ethanone **10b,** using two molar equivalence of bromine in acetic acid at 90–100 ºC, resulted in the formation of bromoacetyl derivative **115**. The structure of **115 **was further confirmed by an independent synthesis outlined in Scheme 36 where compound **116 **treated with equal molar quantity of bromine in acetic acid at 90–100 ºC resulted in the formation of compound **115**. The reaction of compound **115** with thiourea in refluxing ethanol afforded the corresponding 1,3-thiazole derivative **117a**. The reaction of **115** with cyanothioacetamide in refluxing ethanol furnished the cyanomethyl derivative **117b** [73] (Scheme 36). 

When the bromoacetyl **115** was treated with *o*-aminothiophenol in refluxing ethanol, it afforded 2-(2*H*-1,4-benzothiazin-3-yl)-6-bromo-3-methylthiazolo[3,2-*a*]benzimidazole (**118**). Similarly, **115** reacted with *o*-phenylenediamine to afford quinoxaline derivative **119**. Furthermore, treatment of **115** with 2-aminopyridine in refluxing ethanol afforded 6-bromo-2-imidazo[1,2-*a*]pyridin-2-yl-3-methylthiazolo[3,2-*a*]benzimidazole (**120**) [73] (Scheme 36).

#### 3.4.3. Reactions of ethyl 3-methyl-1,3-thiazolo[3,2-*a*]benzimidazole-2-carboxylate (**15a**)

**Scheme 37:**
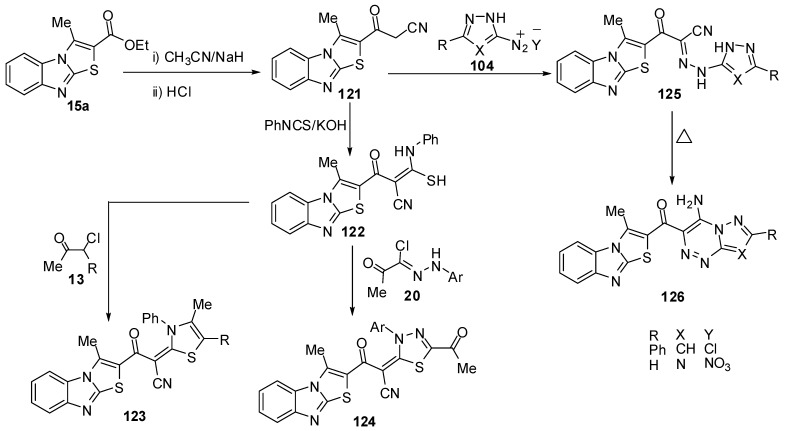
Reaction of ester **15a** with acetonitrile.

3-(3-Methylthiazolo[3,2-*a*]benzimidazol-2-yl)-3-oxopropionitrile (**121**) was synthesized by 3-methylthiazolo[3,2-*a*]benzimidazole-2-carboxylic acid ethyl ester (**15a**), with acetonitrile and sodium hydride [74] (Scheme 37). Treatment of compound **121** with phenyl isothiocyanate, in DMF and in the presence of KOH, at room temperature afforded the non-isolable potassium salt which was converted into the thioacetanilide derivative **122** upon treatment with dilute hydrochloric acid (Scheme 37). Compound **122** reacted with α-chloroacetylacetone (**13a**) and ethyl α-chloroacetoacetate (**13b**) in refluxing ethanol and in the presence of a catalytic amount of triethylamine resulted in the formation of 1,3-thiazole derivatives **123a** and **123b** (Scheme 37). Furthermore, the reaction of thioacetanilide derivative **122** with hydrazonyl chlorides **20** under the same reaction conditions afforded to form 1,3,4-thiadiazole derivatives **124**. On the other hand, treatment of compound **121** with the diazonium salts of both 3-phenyl-5-amino-1*H*-pyrazole **104a** and 5-amino-1*H*-l,2,4-triazole **104b** afforded hydrazones **125a** and **125b** (Scheme 37). Compounds **125a** and **125b** underwent an intramolecular cyclization upon boiling in pyridine *via* Michael type addition of the endocyclic NH of the hydrazones **125a** and **125b** to the triple bond of a nitrile function to afford the corresponding pyrazolo[5,1-*c*]-1,2,4-triazine and 1,2,4-triazolo[5,1-*c*]-1,2,4-triazine derivatives **126a** and **126b, **respectively [74] (Scheme 37).

**Scheme 38:**
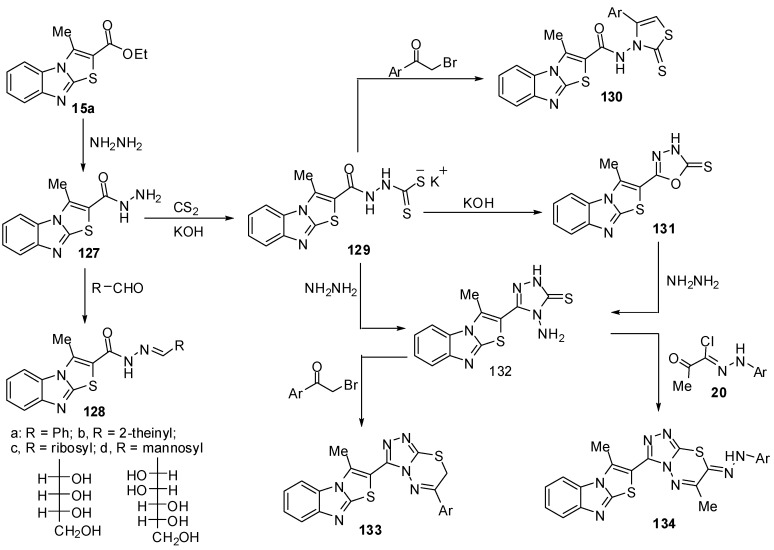
Reaction of ester **15a** with hydrazine hydrate.

Abdel-Aziz *et al.* [75] reported the reaction of 3-methylthiazolo[3,2-*a*]benzimidazole-2-carboxylic acid ethyl ester (**15a**) with hydrazine hydrate in refluxing ethanol to give the hydrazide **127** (Scheme 38). Treatment of compound **127** with the appropriate aldehydes in refluxing ethanol yielded the corresponding hydrazones **128a-d **(Scheme 38). On the other hand, the hydrazide **127** reacts with carbon disulfide in ethanol in the presence of potassium hydroxide to give the potassium salt **129**, which reacts with l-aryl-2-bromoethanones to give the 1,3-thiazolidine derivatives **130**. Heating of the potassium salt **129** in a aqueous solution of potassium hydroxide afforded 5-(3-methylthiazolo[3,2-*a*]benzimidazol-2-yl)-1,3,4-oxadiazole-2-thione (**131**) (Scheme 38). Moreover, treatment of the potassium salt **129** with hydrazine hydrate in a mixture of ethanol and water afforded 4-amino-5-(3-methylthiazolo[3,2-*a*]benzimidazol-2-yl)-4*H*-1,2,4-triazole-3-thione (**132**) [75] (Scheme 38). The structure of compound **132 **was further confirmed by an independent synthesis outlined in Scheme 38. Thus, treatment of 1,3,4-oxadiazole-2-thione derivative **131** with hydrazine hydrate in refluxing ethanol resulted in the formation of a product identical to compound **132**. On the other hand, treatment of compound **132** with l-aryl-2-bromoethanones in refluxing ethanol yielded the 1,2,4-triazolo[3,4-*b*]-1,3,4-thiadiazine derivatives **133**. Similarly, the treatment of triazole**132** with hydrazonyl halides **20 **in ethanol afforded the 1,2,4-triazolo[3,4-*b*]-1,3,4-thiadiazine derivatives **134 **[75] (Scheme 38). 

**Scheme 39:**
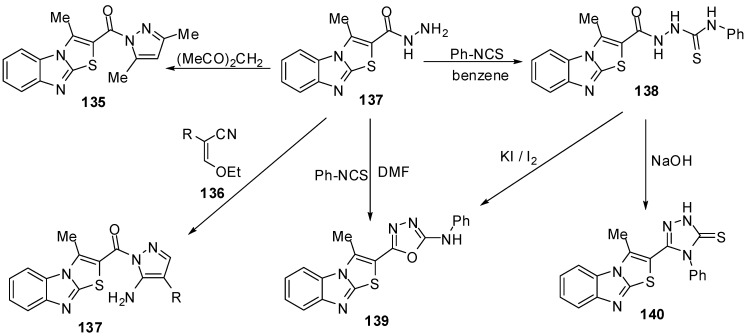
Reactions of hydrazide **137**.

The reaction of hydrazide **127** with pentane-2,4-dione in refluxing ethanol afforded 2-(3,5-dimethylpyrazol-1-oyl)-3-methylthiazolo[3,2-*a*]benzimidazole (**135**), while the reaction of the hydrazide **127** with ethoxymethylene-malononitrile (**136a**) or with ethoxymethylene-ethyl cyanoacetate(**136b**) in ethanol afforded 5-amino-1-(3-methylthiazolo[3,2-*a*]benzimidazol-2-oyl)-1*H*-pyrazole-4-carbonitrile (**137a**) and 5-amino-1-(3-methylthiazolo[3,2-*a*]benzimidazol-2-oyl)-1*H*-pyrazole-4-carboxylic acid ethyl ester (**137b**), respectively [75] (Scheme 39). 

Treatment of the hydrazide **127** with phenyl isothiocyanate in refluxing benzene gave the thiosemicarbazide derivative **138 **[75] (Scheme 39). When the latter reaction of the hydrazide **127 **with phenyl isothiocyanate was carried out in refluxing DMF instead of benzene, the reaction gave 2-(3-methylthiazolo[3,2-*a*]benzimidazol-2-yl)-5-phenylamino-1,3,4-oxadiazole (**139**). On the other hand, the structure of compound **139 **was further confirmed by an independent synthesis by treatment of the thiosemicarbazide derivative **138** with potassium iodide and iodine in the presence of sodium hydroxide. Furthermore, The intramolecular cyclization of thiosemicarbazide derivative **138** takes place upon heating with sodium hydroxide to produce the 1,2,4-triazole derivative **140 **[75] (Scheme 39).

**Scheme 40:**
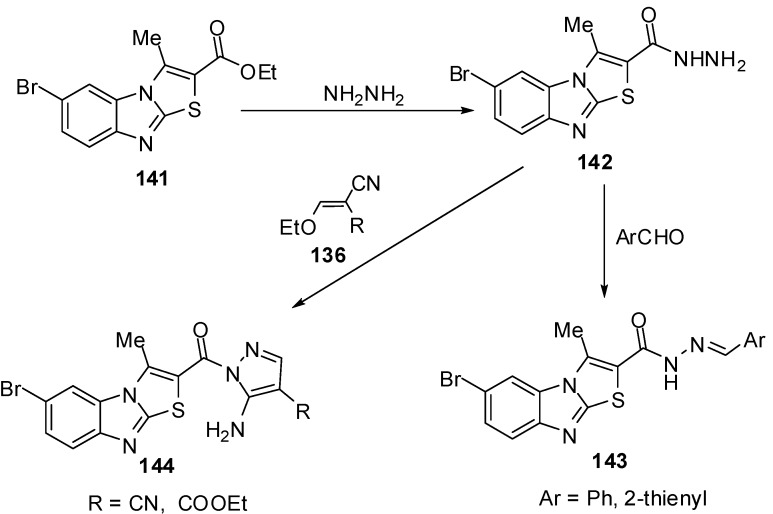
Reactions of hydrazide **142**.

Abdel-Azia *et al.* [76] reported the synthesis of 6-bromo-3-methylthiazolo[3,2-*a*]benzimidazole-2-carboxylic acid ethyl ester (**141**) by the bromination of ester **15a**. Compound **141** reacted with hydrazine hydrate in refluxing ethanol to give the hydrazide **142** (Scheme 40). The treatment of compound **142** with benzaldehyde or 2-thiophenaldehyde, in refluxing ethanol yielded the corresponding hydrazones **143a** and **143b**, respectively. On the other hand, the reaction of hydrazide **141** with ethoxymethylene malononitrile (**136a**) or with ethyl ethoxymethylene cyanoacetate (**136b**) in ethanol afforded pyrazole derivatives **144a** and **144b**, respectively [76] (Scheme 40).

#### 3.4.4. Reactions of thiazolo[3,2-*a*]bezimidazol-3(2*H*)ones **2**

**Scheme 41:**
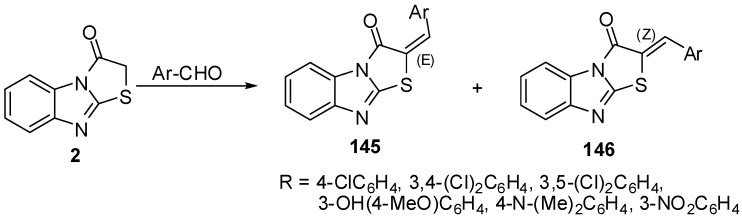
Reaction of thiazolone **2** with aromatic aldehydes.

Refluxing of thiazolo[3,2-*a*]benzimidazol-3(2*H*)-one (**2**) with aromatic aldehydes in pyridine/ dicyclohexylcarbodiimide, EtOH/piperidine or in AcOH/AcONa, gave *E/Z* 2-arylidenethiazolo[3,2-*a*]benzimidazol-3(2*H*)-ones **145/146 **[77,78,79] (Scheme 41).

The condensation of thiazolones **2** with 1,3-benzodioxole-5-carbaldehyde (**147**) and indole-3-carbaldehyde (**148**), using pyridine as a catalyst resulted in products **149** and **150**, respectively. 

**Scheme 42:**
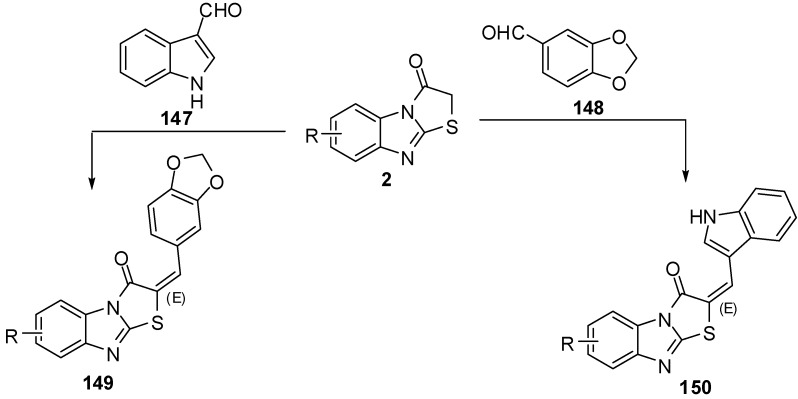
Reaction of thiazolone **2** with aldehydes.

The one-pot synthesis of these compounds carried out by a cyclocondensation (a Knoevenagel condensation, followed by cyclization) of compounds **3**, chloroacetic acid, aromatic or heteroaromatic aldehyde, acetic anhydride, and glacial acetic acid in the presence of sodium acetate (piperidine) led to 2-substituted thiazolo[2,3-*a*]benzimidazole-3(2*H*)-ones **149** and **150** in good yields [80] (Scheme 42). 

**Scheme 43:**
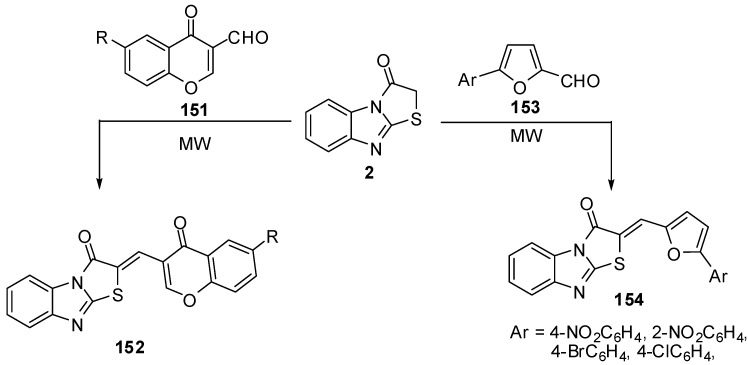
Reaction of thiazolone **2** with aldehydes.

Condensation of 6-*R*-3-formylchromones **151** with thiazolone **2** by the classica method, as well as condensation in a microwave oven to synthesis compounds **152**, has been studied [81] (Scheme 43). Synthesis of compounds **154** from 5-arylfuran-2-carboxaldehydes **153** have been studied in acetic anhydride, by both classical heating and under microwave assisted conditions. The beneficial effect of microwave irradiation on these condensations was found in a shortening of the reaction time and increase in the yields [82] (Scheme 43).

**Scheme 44:**
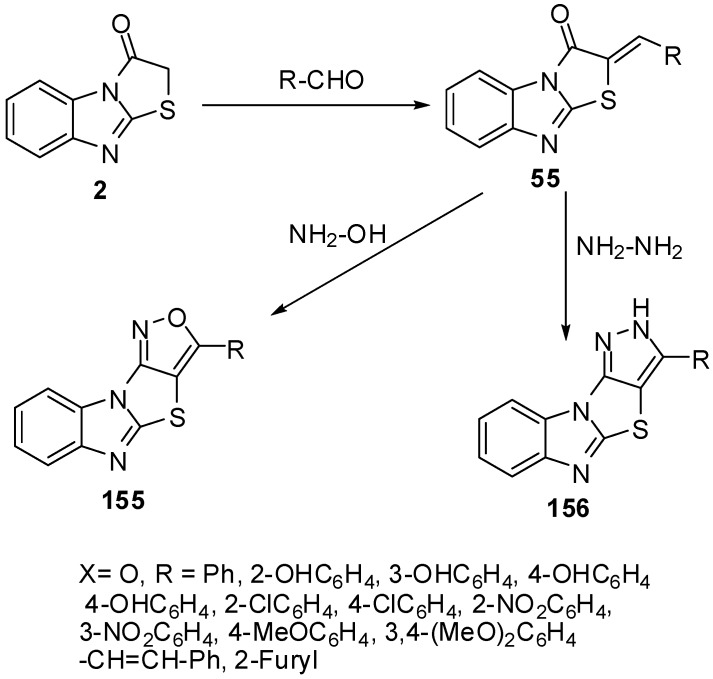
Reaction of thiazolone **2** with aldehydes.

Several fused isoxazoles **155** were synthesized by the reaction of hydroxyl amine with benzylidines derivatives **55 **[83] (Scheme 44). 

On the other hand, dihydropyrazoles **156** were synthesized by condensation of hydrazine hydrate with arylidines **55** under microwave irradiation and solvent free conditions [84] (Scheme 44).

**Scheme 45:**
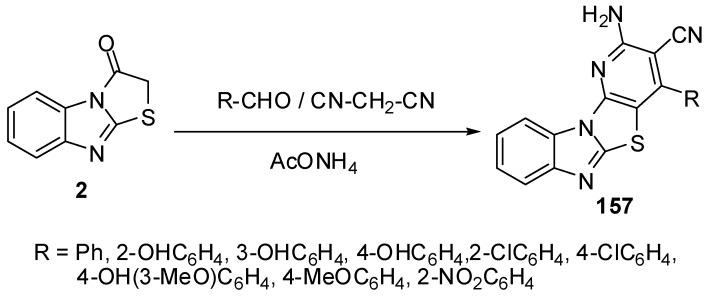
One-pot reaction of pyridines **157**.

Furthermore, condensed pyridines **157** were prepared in one-pot three component reaction from thiazolone **2**, aromatic aldehydes and malononitrile [83] (Scheme 45).

**Scheme 46:**
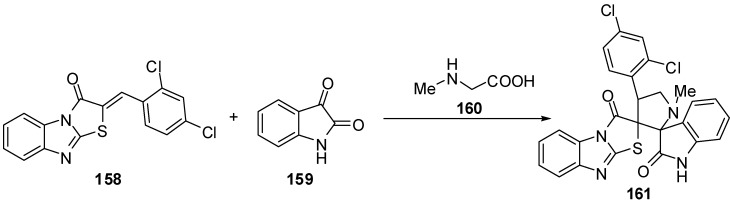
Synthesis of spiro-pyrrolidine **161**.

4'-(2,4-Dichlorophenyl)-1'-methyl-2,3,2'',3''-tetra-hydro-1*H*-indole-3-spiro-2'-pyrrolidine-3'-spiro-2'-(1,3-benzimidazo[2,1-*b*]thiazole)-2,3''-dione **161** was synthesized by the intermolecular [3+2]-cycloaddition of azomethine ylide, derived from isatin **159** and sarcosine **160** by a decarboxylative route, and 2-(2,4-dichlorobenzylidene)benzo[4,5]imidazo[2,1-*b*]thiazol-3-one **158 **[85] (Scheme 46).

**Scheme 47:**
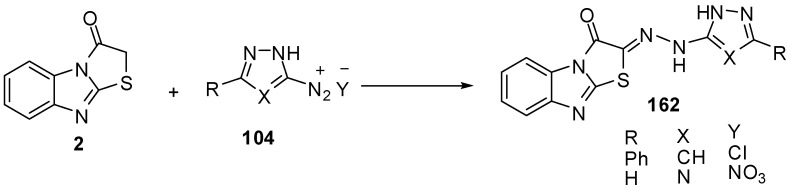
Coupling reaction of thiazolone **2**.

Compound **2** couples smoothly with 3-phenyl-1*H*-pyrazole-5-diazonium chloride (**104a**) and 1*H-*l,2,4-triazole-5-diazonium nitrate (**104b**) to afford the corresponding hydrazones **162a** and **162b **[74] (Scheme 47). 

**Scheme 48:**
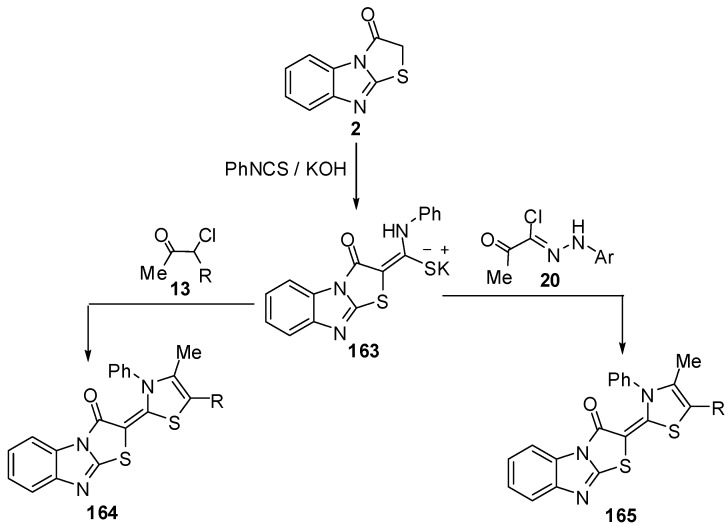
Reaction of thiazolone **2** with phenyl isothiocyanate.

Treatment of thiazolo[3,2-*a*]benzimidazol-3(2*H*)-one (**2**) with phenyl isothiocyanate, in DMF and in the presence of KOH, at ambient temperature furnished the nonisolable potassium salt **163** which reacts *in situ* with α-chloroacetylacetone (**13a**) and ethyl α-chloroacetoacetate (**13b**) to give 1,3-thiazole derivatives **164a** and **164b**. In a similar manner, hydrazonyl chlorides **20** reacted with the non-isolable potassium salt **163** under the same reaction conditions, to afford 1,3,4-thiadiazole derivatives **165 **[74] (Scheme 48). 

**Scheme 49:**
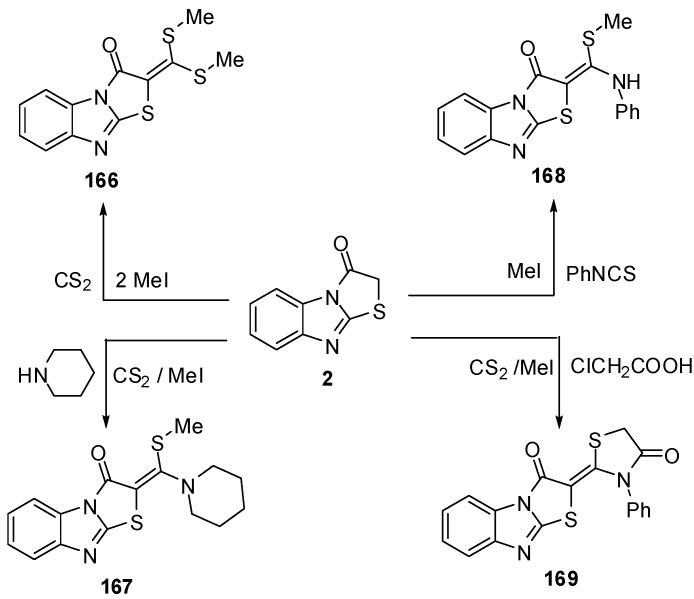
Reaction of thiazolone **2** with carbon disulphide.

Treatment of thiazolone **2** with carbon disulphide followed by the reaction with methyl iodide or piperidine afforded compounds **166** and **167**, respectively, while the reaction of thiazolone **16** with pheny isothicyanate and methyl iodide or chloroacetic acid gave compounds **168** and **169**, respectively [79] (Scheme 49).

### 3.5. Reactions of C2-C3 [Reactions of 3-aminothiazolo[3,2-*a*]benzimidazol-2-carbonitrile (**41**)]

**Scheme 50:**
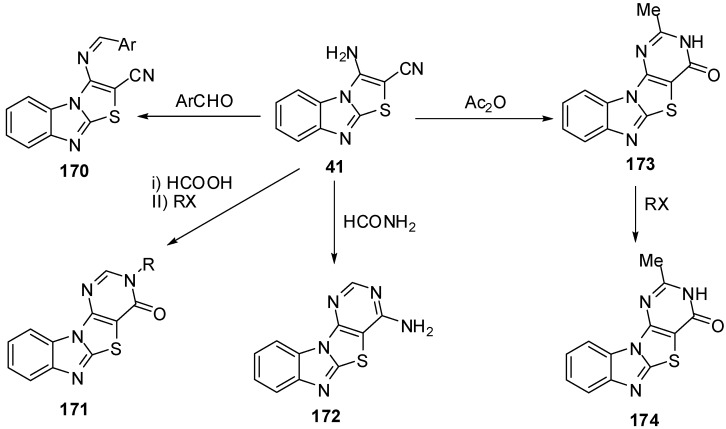
Reactions of 3-aminothiazolo[3,2-*a*]bezimidazole-2-carbonitrile **41**.

Synthesis and reactions of 3-aminothiazolo[3,2-*a*]bezimidazole-2-carbonitrile **41** were reported by Sarhan and his co-workers [11,47,48,86,87,88,89]. Condensation of compound **41** with aromatic aldehydes afforded the arylmethylidenes **170**. Cycliztion of **41** with formic acicc, formamide and acetic anhydride gave fused pyrimidines **171**, **172** and **173**, respectively. Alkylation of **173** with alkyl halides gave the *N*-alkyl derivatives **174** (Scheme 50).

**Scheme 51:**
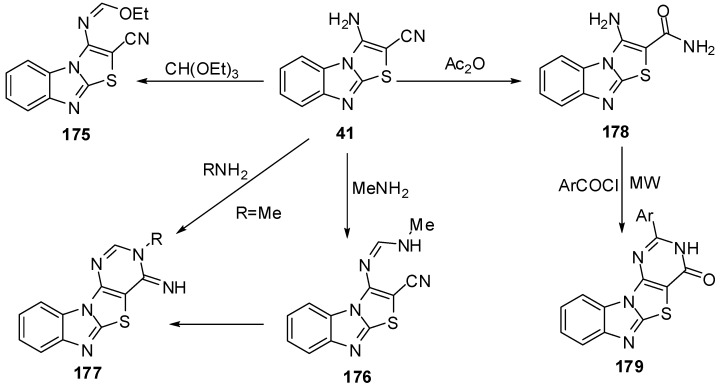
Reactions of 3-aminothiazolo[3,2-*a*]bezimidazole-2-carbonitrile **41**.

Reaction of **41** with triethyl ortho formate gave the ethoxymethyleneamino derivative **175**, which cyclized with different amines to give the products **176** and **177**. Hydrolysis of **41** by H_2_SO_4_ or H_3_PO_4_ afforded the amide derivative **178** (Scheme 51).

On the other hand, Davoodnia *et al.* [90] reported the reaction of compound **178** with aroyl halides under microwave irradiation in solvent-free condition at 800 W to afford 2-arylpyrimido[4',5':4,5]-thiazolo[3,2-*a*]benzimidazol-4(3*H*)-ones **179** (Scheme 51).

**Scheme 52:**
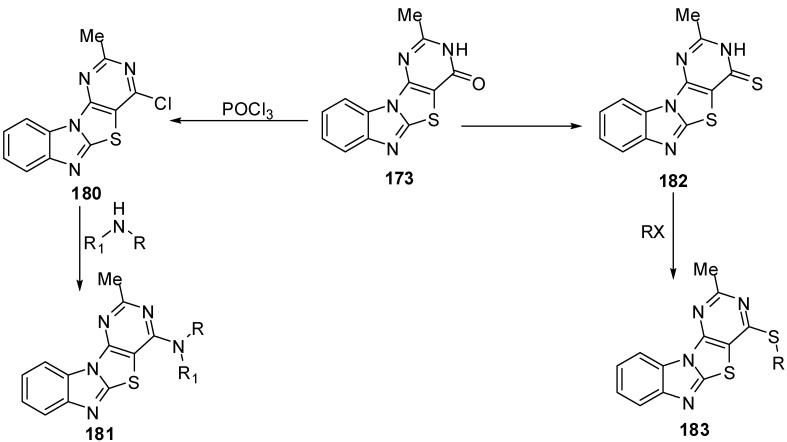
Reactions of pyrimidinone **173**.

4-Chloro-2-methylpyrimidino[4',5':4,5]-thiazolo[3,2-*a*]benzimidazole (**180**) was prepared by chlorination of pyrimidine **173**. Nucleophilic substitution of **180** with alcohols, phenols, primary amines, secondary amines, sodium azide, and mercaptoacetic acid gave the corresponding derivatives **181**. Thination of fused pyrimidine **173** afforded 2-methylpyrimidino[4',5':4,5]-thiazolo[3,2-*a*]benzimidazol-4-thiol (**182**). The thiol derivative **182** was reacted with alkyl/aralkyl halides, phenacyl bromide derivatives, bromoacetone, chloroanilides, bromomalonic ester, and ethyl bromoacetate to afford sulphides **183** [88] (Scheme 52).

**Scheme 53:**
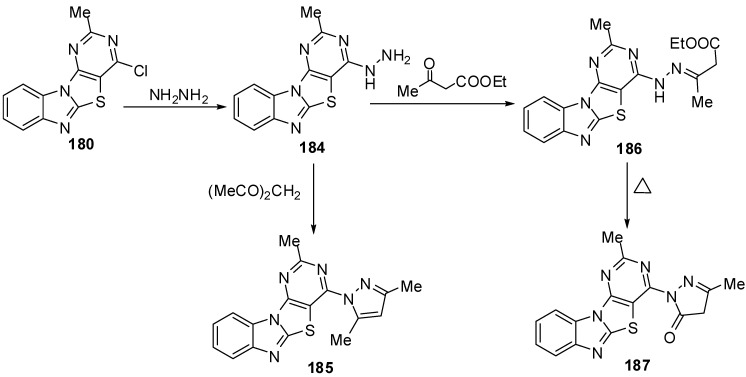
Reactions of chloro pyrimidine **180**.

In addition, the reaction of chloro compound **180 **with hydrazine hydrate in refluxing ethanol gave 4-hydrazino-2-methylpyrimidino[4',5':4,5]thiazolo[3,2-*a*]benzimidazole (**184**) as showed in Scheme 53. Condensation of 4-hydrazino derivative **184 **with acetyl acetone under neat conditions gave the corresponding 2-methyl-4-(3,5-dimethylpyrazolyl)pyrimidino[4',5':4,5]-thiazolo[3,2-*a*]benzimidazole (**185**) [88] (Scheme 53). 

Condensation of **184 **with ethyl acetoacetate in refluxing ethanol afforded the uncyclized derivative **186 **which, on heating over its melting point, resulted in the formation pyrazolone derivative **187 **[88](Scheme 53).

The reaction of hydrazino derivative **184** with triethyl orthoformate gave the corresponding 4-ethoxymethylidenehydrazino-2-methylpyrimidino[4',5':4,5]-thiazolo[3,2-*a*]benzimidazole (**188**). On heating of **188** under neat conditions afforded the triazolo[2,3-*c*] isomer **189**. In addition, the triazolo[2,3-*c*] isomer **189** was also obtained by refluxing **184** in formic acid or formic acid/glycerol mixture at refluxing temperature (Scheme 53). 

**Scheme 54:**
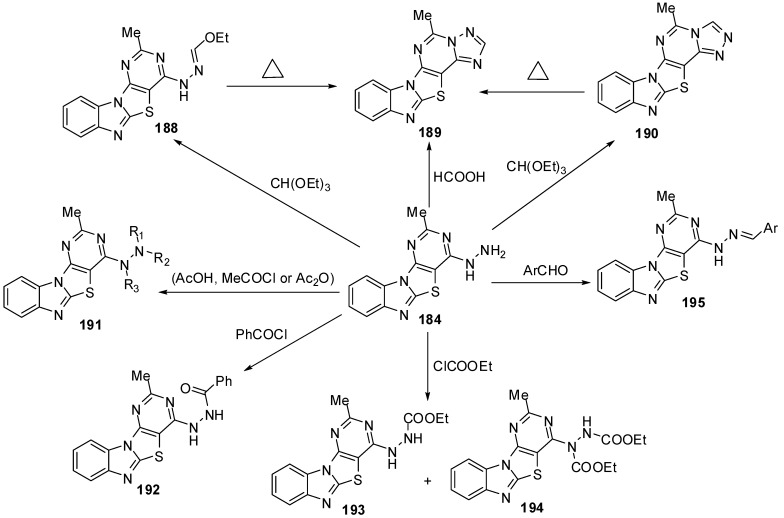
Reactions of hydrazino pyrimidine **184**.

Refluxing the hydrazino compound **184** with triethyl orthoformate gave the triazolo[4,3-*c*] isomer **190** which was isomerized on heating over its melting point to give the isomer **189**. The triazolo[2,3-*c*] derivatives undergo isomerization to [3,4-*c*] isomers [88] (Scheme 54).

The reaction of **184** with acetic acid afforded the mono acetyl derivative **191a**. While, when acetyl chloride was used as acetylating agent the diacetyl derivative **191b** was obtained be sides the triacetyl derivative **191c**. Moreover the triacetyl derivative **192c** was obtained independently in pure form by refluxing **184** with acetic anhydride. Benzoylation of the hydrazino compound **184** was carried out in CHCl_3_ containing K_2_CO_3_ with benzoyl chloride to afford the mono benzoyl derivative **192**. Reaction of **184** with ethylchloroformate afforded a mixture of separable carbamates **193** and **194**. Moreover, condensation of hydrazino derivative 184 with aromatic aldehydes obtained the *E*-form of arylmethylideneamino derivatives **195** [88] (Scheme 54). 

### 3.6. Reactions of C3

**Scheme 55:**
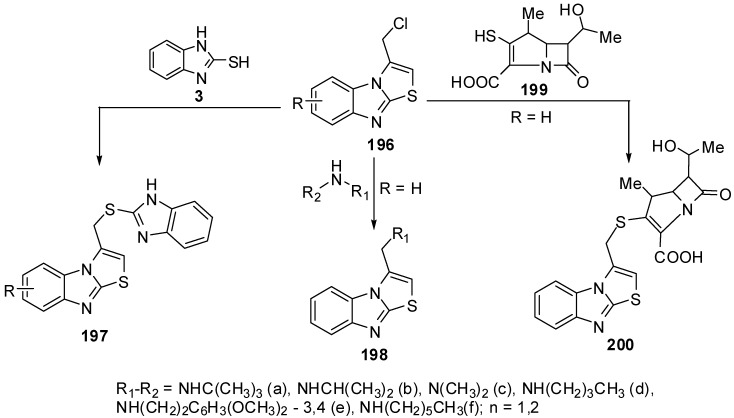
Reactions of 3-chloromethythiazolo[3,2-*a*]benzimidazole (**196**).

Compounds **197** were prepared from the reaction of 3-chloromethythiazolo[3,2-*a*]benzimidazole (**196**) with 2-mercaptobenzimidazole **3 **[91], while 3-aminomethylthiazolo[3,2-*a*]benzimidazoles **198** were synthesized by reacting 3-(chloromethyl)thiazolo[3,2-*a*]benzimidazole **196** with primary and secondary amines [92] Recently, *β*-methyl carbapenem incorporating thiazolobenzimidazole moiety compound **200 **[93] was prepared from lactame **199** (Scheme 55).

### 3.7. Reactions of C3-N4

**Scheme 56:**
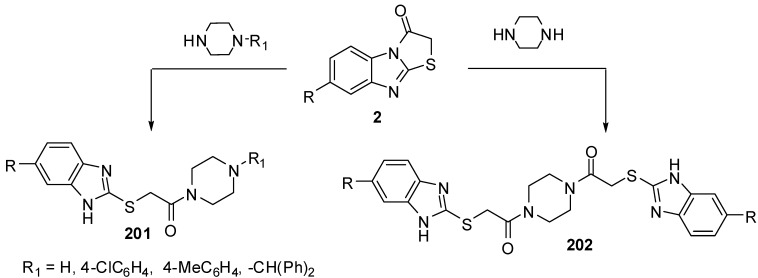
Reactions of thiazolones **2** with piperazines.

The hydrolysis of thiazolo[3,2-*a*]benzimidazol-3(2*H*)-ones **2** with piperazine or an appropriated *N*-monosubstituted piperazine in ethanol under refluxing conditions resulted in the formation of piperazine derivatives **201 **and **202**, respectively [94] (Scheme 56).

### 3.8. Reactions of C6

**Scheme 57:**
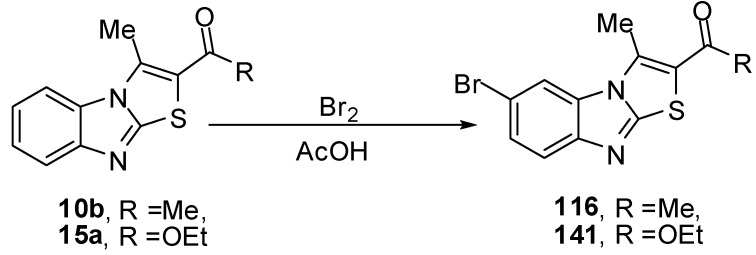
Bromination of ethanone **10b **and ester **15a**.

Compound **116** was prepared from ethanone **10b** and bromine in acetic acid at ambient temperature [76]. Compound **141** was prepared by the treatment of 1-(3-methylthiazolo[3,2-*a*]benzimidazol-2-yl)ethanone (**15a**) under the same reaction conditions. The structure **141** was assigned for the reaction product on the basis of its single crystal X-ray diffraction [73] (Scheme 57).

### 3.9. Reactions of N9

**Scheme 58:**
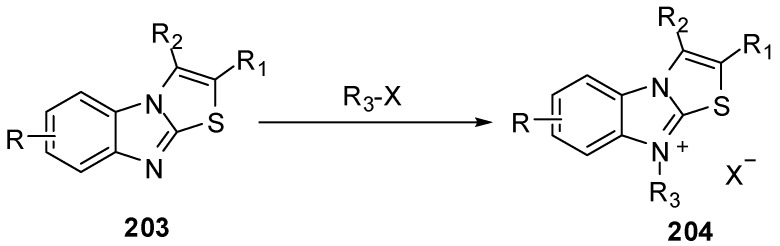
Reaction of compounds **203** with alkyl or aryl halides.

Treatment of compounds **203** with alkyl or aryl halides [95] gave 9-substituted thiazolo[3,2-*a*]benzimidazolium salts **204** (Scheme 58).

### 3.10. Reactions of N9-C9a

**Scheme 59:**
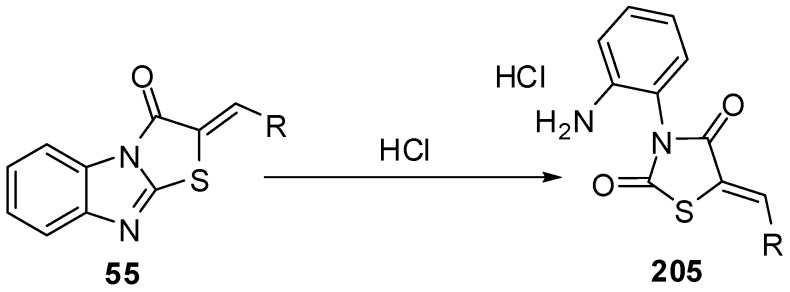
Acid hydrolysis of arylidene derivatives **55**.

Acid hydrolysis of arylidene derivatives **55** lead to the formation of hydrochlorides of the corresponding thiazolid-2,4-ones **205***via* the rupture of endocyclic C=N bonds in imidazole ring [96,97] (Scheme 59). 

## 4. Spectral characteristics

Recently, Abdel-Aziz *et al.* reported the single crystal X-ray diffractions of 2-[2-(4-methoxyphenyl)pyrazolo[1,5-*a*]pyrimidin-7-yl]-3-methylthiazolo[3,2-*a*]benzimidazole **95b **[71] and 1-(6-bromo-3-methylthiazolo[3,2-*a*]benzimidazol-2-yl)ethanone **141** [73]. Crystallography studies of 4'-(2,4-dichlorophenyl)-1'-methyl-2,3,2'',3''-tetra-hydro-1*H*-indole-3-spiro-2'-pyrrolidine-3'-spiro-2'-(1,3-benzimidazo[2,1-*b*]thiazole)-2,3''-dione **161** [85], 4-(2-chlorophenyl)-3-(2,6-dichlorophenyl)-spiroisoxazoline-5,2`-thiazolo[3,2-*a*]benzimidazol-3(2*H*)-one dioxane hemisolvate [98] and 5`-(2-chlorophenyl)-1`-methyl-2"-(thiazolo[3,2-*a*]benzimidazol-3(2*H*)-one)-2,3"-dione dioxane hemisolvate [99] were reported. The electron impact mass spectra of *β*-D-glucopyranuronic acid of thiazolo[3,2-*a*]benzimidazole derivatives [100] and IR spectra of some thiazolo[3,2-*a*]benzimidazole derivatives [101] were reported. 

## 5. Biological Activities

This section highlights the biological activities of thiazolo[3,2-*a*]benzimidazoles published in the last 22 years, as other biological activity appeared up to 1988 were collected in the review of Chimirri *et al*. [9]. Diverse biological properties have been associated with thiazolo[3,2-*a*]benzimidazole derivatives in last two decades, including antibacterial [77,93,102], antifungal [69], anti-inflammatory [95,103], antiulcer [104,105,106,107], antiviral [108,109], anthelmintic [18,110] and anticancer activity [71,73,76]. The parasitological study *in vitro* showed that the heterocyclic benzylidines of thiazolo-[3,2-*a*]benzimidazoles exhibited higher activity than albendazole against *T. spiralis* [80].

Moreover, thiazolo[3,2-*a*]benzimidazole derivatives are well known as platelet activating factor antagonists [111] and neoplasm inhibitors [112]. Some thiazolo[3,2-*a*]benzimidazole derivatives inhibit H+/K+-ATPase and gastric secretion and are thus useful as antiulcer agents [113]. Furthermore, thiazolo[3,2-*a*]benzimidazol-1-oxide (WY-26,769) shows gastric antisecretory activity [114].

3-Amino-derivatives of thiazolobenzimidazole inhibited, to different extents, the oxidation of adrenaline to adrenochrome, thus preventing formation of the superoxide radical [92]. Some synthesized thiazolobenzimidazoles showed antiparasitic activity on the helminth *Trichinella spiralis* in infected white mice *in vitro* as well as *in vivo* [115]. In addition, many thiazolo[3,2-*a*]benzimidazole derivatives are of great importance due to their antidiabetic [16], broncholytic [91], immunotropic [17] and antitrichomonal activities [116]. On the other hand, several thiazolo[3,2-*a*]benzimidazole derivatives are used for cancer treatment [117] or prevention of cerebral infarction [118], and the treatment and/or prevention of bone diseases [119].

**Figure 3:**
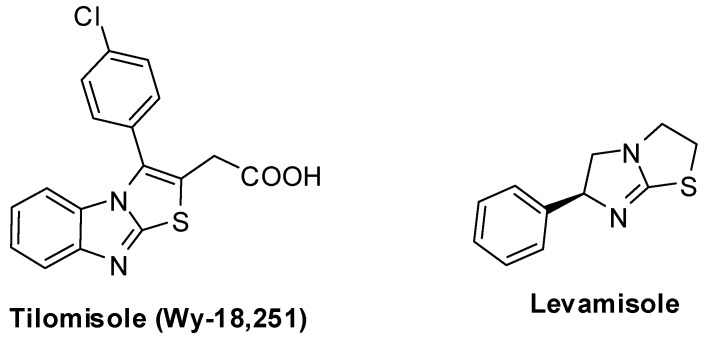
Chemical structure of tilomisole (WY-18,251) and levamisole.

Tilomisole (WY-18,251) [120,121,122,123,124,125,126,127,128] (Figure 3) has been widely studied. It showed several potent activities, such as antinflammatory activity [123,124,125,126]. It has been reported to possess remarkable anticancer activity since it could be considered an analog of levamisole, a well-known immunomodulator which is used for the adjuvant treatment of the colon cancer. However, tilomisole (Wy-18,251) has favorable biological response effects *in-vivo* and it is a suitable alternative to levamisole in cancer treatments [127,128]. 

The combination effect of tilomisole (Wy-18,251) with aspirin or naproxen was studied in rats with carrageenan-induced paw edema and established adjuvant arthritis was reported [126]. Tilomisole was found effective in inhibiting of alpha-interleukin 1 (IL-1)-induced cartilage proteoglycan resorption *in-vitro* [129].

**Figure 4:**
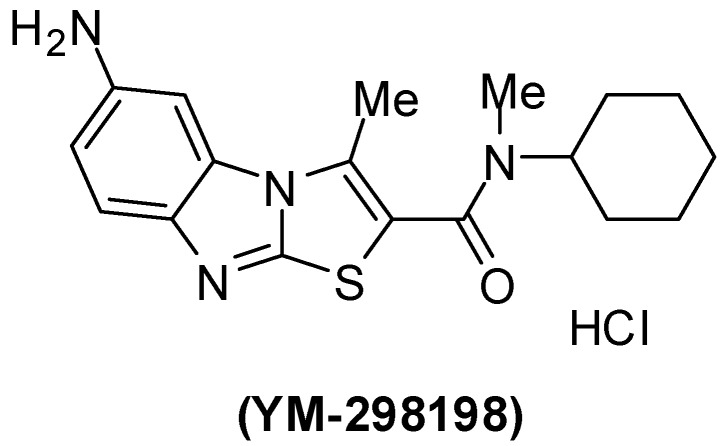
Chemical structure of YM-298198.

Pharmacological characterization of 6-amino-*N*-cyclohexyl-*N*,3-dimethylthiazolo[3,2-*a*]benzimidazole-2-carboxamide (YM-298198) (Figure 4), a high-affinity, selective, and noncompetitive antagonist of metabotropic glutamate receptor type 1 was reported [130,131,132,133,134].

The mGlu1 antagonist YM-298198 in a physiological functional assay facilitates elucidation of this receptor's role in brain function and as a potential drug target. This compound is more potent than previously available compounds [131,132,133]. However, YM-298198 represented as the most potent known blocker of type I mechanoreceptors [134]. It also used in the treatment of neurogenic pain [135].

**Figure 5:**
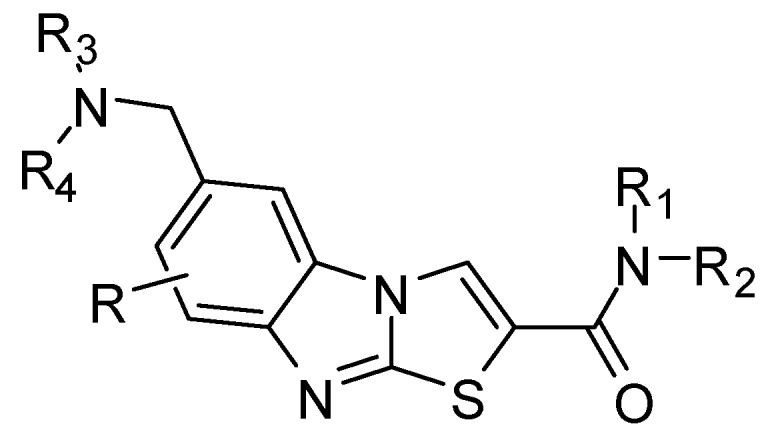
6-Aminomethyl-substituted thiazolobenz­imidazole derivatives.

6-Aminomethyl-substituted thiazolobenz­imidazole derivatives (R = H) (Figure 5) act as remedies for schizophrenia [136]. These compounds used in treatment or prevention of mGluR1 related diseases [137] (epilepsy, inhibition of nerve cell death, Parkinson's disease, migraine headache, anxiety disorder, cerebral infarction and neurogenic pain). 6-Aminomethyl-substituted fluorothiazolobenz­imidazole derivative (R = F) as a metabotropic glutamate receptor, has excellent activity in oral administration, and is useful as a medicine [138]. 

The activities of thiazolo[3,2-*a*]benzimidazole derivatives also extend into fields other than the medicinal one; for example, they are used in magnetic recording disks [139] and in photographic fields [140,141,142].

## 6. Conclusions

In light of the literature reports cited herein, the synthetic strategies and subsequent chemical transformations of the resulting thiazolo[3,2-*a*]benzimidazoles provides several important classes of functionalized compounds. The simplicity and flexibility of the experimental procedures in the generation of these classes, together with the diversity of thiazolo[3,2-*a*]benzimidazole chemistry, make these synthetic methodologies a highly efficient and practical method for preparation of various biologically active derivatives. The investigations in the pharmaceutical filed and medicinal applications are developing quite rapidly and we hope it will bring new and useful results.
